# COVID-19-associated cutaneous vasculitis with exfoliative erythroderma and extensive scarring: Long-term clinical and laser management

**DOI:** 10.1016/j.jdcr.2026.04.071

**Published:** 2026-05-15

**Authors:** Britney T. Nguyen, Xiying Fan, Kenneth G. Linden, Nathan Rojek, Shireen V. Guide, Andrew I. Shulman, Kristen M. Kelly, Bonnie A. Lee

**Affiliations:** a Department of Dermatology, University of California Irvine, Irvine, California; b Mission Dermatology Center, Rancho Santa Margarita, California; c Department of Dermatology, Children’s Hospital of Orange County, Orange, California; d Division of Rheumatology, Children’s Hospital of Orange County, Rady Children’s Health, Department of Pediatrics, University of California Irvine, Orange, California

**Keywords:** COVID-19, cutaneous vasculitis, hypertrophic scar, laser, overlap syndrome, psoriasiform dermatitis

## Introduction

Infection with the coronavirus disease 2019 (COVID-19) can result in various cutaneous manifestations,^1^ including onset or exacerbation of autoimmune and inflammatory diseases.^2^ We report a case of post-COVID-19 cutaneous vasculitis in a fully immunized twelve-year-old girl with prolonged hospitalization for diffuse desquamative erythroderma and substantial ulceration resulting in hypertrophic scarring. We summarize her unique course and long-term outcomes, including successful laser scar treatment without disease flare.

## Case

**Fig 1:**
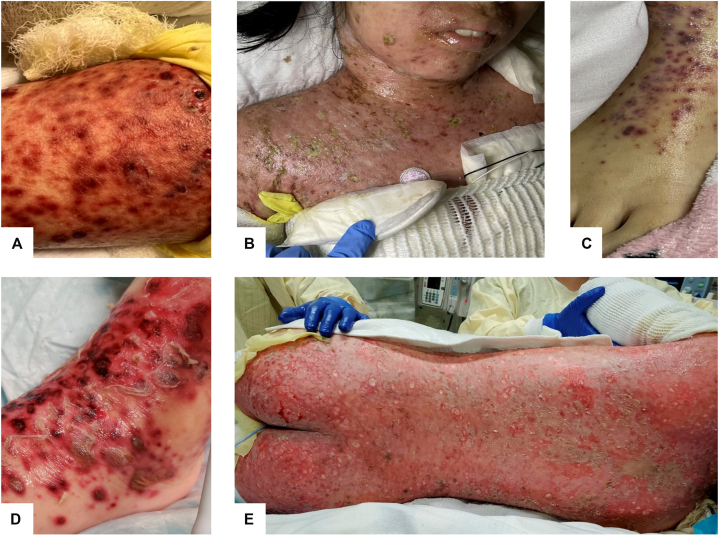
Centrifugal progression of ulcerating purpuric papules after COVID-19 infection. **A,** Initial presentation showing the proximal thigh on hospital day 2 (HD2). **B** and **C,** By HD8, the eruption had spread acrally and progressed to ulceration axially. Note lack of involvement of the lips. **D** and **E,** On HD16, epidermal sloughing on the dorsal foot and widespread ulceration on the trunk. *HD*, Hospital day.

Five days after testing positive for COVID-19, a healthy twelve-year-old girl presented to an outside facility with widespread painful, ulcerating, purpuric papulonodules with desquamation (Fig 1, *A*-*C*), mild upper respiratory symptoms, and no mucosal involvement. Outpatient oral nonsteroidal anti-inflammatory drugs, topical steroids, and a short prednisone course yielded no improvement. Her parents adamantly denied preceding medications, travel, or personal/family history of dermatologic disease, autoimmunity, or malignancy.

**Fig 2:**
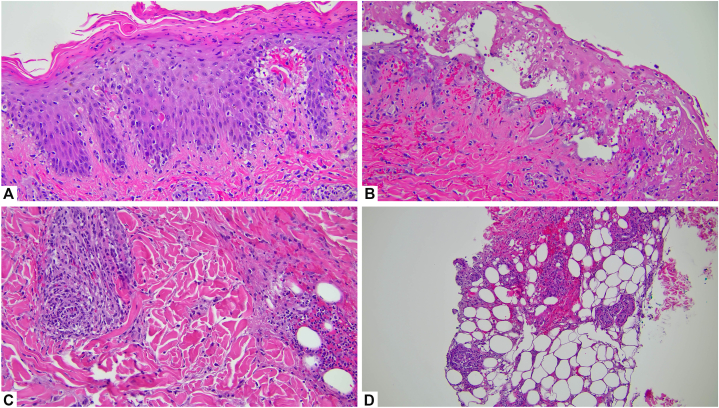
Histopathology of cutaneous vasculitis. **A,** Interface dermatitis with scattered necrotic keratinocytes and superficial epidermal necrosis (200× magnification). **B,** Focal area of full-thickness epidermal necrosis (200× magnification). **C,** Small-vessel vasculitis with fibrinoid necrosis in the mid-dermis (200× magnification). **D,** Medium-vessel vasculitis in the subcutaneous tissue (100× magnification).

On admission to our children’s hospital, labwork revealed elevated transaminases (aspartate aminotransferase 65, alanine aminotransferase 65 units/L), C-reactive protein (8.9; reference 0.0-1.0 mg/dL), erythrocyte sedimentation rate (74 mm/h) and positive COVID-19 polymerase chain reaction. Blood counts, creatine kinase, and troponin were unremarkable. Streptococcal rapid antigen testing was positive, but a throat culture was negative. Wound cultures grew methicillin-sensitive *Staphylococcus aureus*; urine culture grew *Proteus mirabilis*, *S aureus*, and *E*. *coli*. Herpes simplex virus, interferon-γ release assay, parvovirus, cytomegalovirus, and typhus were negative. A skin biopsy showed small- and medium-vessel vasculitis and interface dermatitis with focal full-thickness epidermal necrosis, suggesting a cytotoxic viral-associated immune response resembling Stevens-Johnson syndrome (SJS)/toxic epidermal necrolysis (TEN) (Fig 2, *A*-*D*) superimposed on vasculitis. Direct immunofluorescence studies were negative, militating against an autoimmune blistering disease.

Cefazolin and clindamycin were initiated empirically for staphylococcal scalded skin syndrome, then broadened to cefepime, oxacillin, and fluconazole/micafungin for new vasculitic lesions and recurrent fevers. She received intravenous immune globulin (IVIG) and methylprednisolone. After 2 weeks, her skin disease stabilized (Fig 1, *D* and *E*), involving ∼80% body surface area with dark red-purple ulcerating papulonodules with widespread desquamation, sparing mucosa.

After transfer to our burn center, she underwent a 3-wk methylprednisolone taper and a 7-day empiric course of intravenous acyclovir, despite negative lesional herpes simplex virus/VZV polymerase chain reaction. Anti-streptolysin O, anti-DNase B, anti-neutrophil cytoplasmic antibodies, anti-double-stranded DNA, rheumatoid factor, cryoglobulins, and complements were unremarkable. After 1 month of burn care, her skin finally re-epithelialized, and she was discharged.

**Fig 3:**
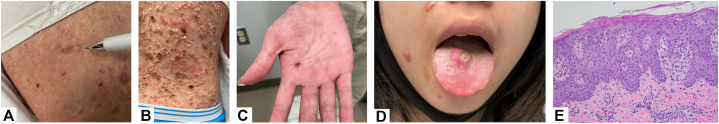
Evolution of psoriasiform dermatitis with contemporaneous reemergence of violaceous lesions after hospital discharge. **A** and **B,** Diffuse psoriasiform dermatitis shown on lower back at **(A)** 14 and **(B)** 16 weeks after hospital. **C** and **D,** Rare ulcerating purpuric papules. **E,** Punch biopsy at 200× magnification showing epidermal hyperplasia, parakeratosis, and hypogranulosis with mild spongiosis and few necrotic keratinocytes.

At 1-month follow-up, she had widespread post-inflammatory hyperpigmentation and hypertrophic/atrophic scarring, few scattered purpuric papulonodular ulcers, and a new, diffuse papulosquamous eruption (Fig 3, *A*-*D*). A biopsy of the latter demonstrated psoriasiform epidermal hyperplasia with foci of superficial epidermal necrosis (Fig 3, *E*). Her oral mucosa remained clear of desquamative disease, though a solitary ulcerated nodule developed on the tongue. We favored that the constellation of findings represented a milder, more chronic phase of her initial disease. Topical steroids and secukinumab cleared the psoriasiform eruption, though malaise, low-grade fevers, elevated erythrocyte sedimentation rate (106 mm/h), and 1-3 new purpuric ulcers weekly persisted. Cyclosporine and prednisone were initiated, transitioned to cyclosporine plus IVIG, then IVIG alone, with complete resolution of skin and constitutional symptoms by 6 months postdischarge.

**Fig 4:**
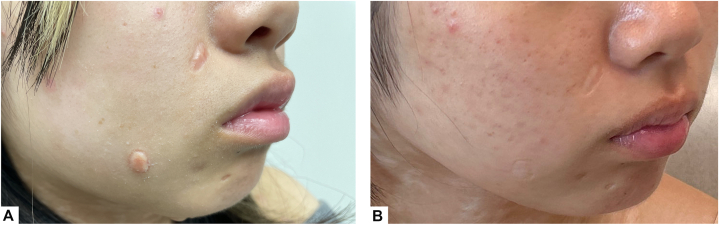
Long-term right cheek outcomes. **A,** Residual scarring and postinflammatory hyperpigmentation about 1 year after initial presentation. **B,** Significant improvement in hypertrophic scarring after laser therapy.

One year after presentation, her disease remained in remission on monthly IVIG (Fig 4, *A*). She began pulsed-dye laser (10 mm spot size; 1.5 ms pulse duration, 5.0-5.5 J/cm^2^, cryogen cooling with 30 ms spurt, and 30 ms delay) and fractional ablative erbium:YAG (2940 nm, 300-350 μm depth, 5.5% density, air cooling) treatment along with topical (laser-assisted drug delivery) and/or intralesional triamcinolone 40 mg/cc to the hypertrophic scars. After 2 years, IVIG was tapered and discontinued. Three years out and off immunomodulators, she has had substantial scar improvement without disease flare (Fig 4, *B*).

## Discussion

This case highlights the unusual combination of a diffusely ulcerative cutaneous small and medium-vessel vasculitis with widespread exfoliative erythroderma sparing the mucosa, and a convalescent psoriasiform eruption in an otherwise mild systemic COVID-19 infection. A possible hypothesis is that COVID-19 triggered concurrent vascular and epidermal injury. Prior studies have demonstrated SARS-CoV-2 spike proteins within dermal microvascular endothelial cells, supporting antigen-driven endothelial activation and injury.^3^ Although vasculitic and vasculopathic complications of COVID-19 are recognized,^2^^,^^4^^,^^5^ to our knowledge, none share these unusual histopathologic and clinical features.

The exfoliative erythroderma mimicked SJS/TEN yet spared the mucosa and lacked preceding medication exposure, rendering SJS/TEN less likely. Reports of COVID-19-associated cutaneous eruptions demonstrate interface dermatitis and keratinocyte death, even in clinically typical viral exanthems, suggesting epidermal injury may occur in response to COVID-19.^3^^,^^6^ We hypothesize that our patient’s desquamative erythroderma, which required 2 months of hospitalization, may represent an extreme COVID-associated viral exanthem, superimposed on the more commonly reported vasculitic eruption,^7^ and her convalescent psoriasiform phase represents reactive epidermal hyperplasia following chronic epidermal injury and/or interface dermatitis.

This uncertain overlap initially prompted consideration of Kawasaki disease and multisystem inflammatory syndrome in children (MIS-C). However, she lacked cardiopulmonary or gastrointestinal involvement seen in MIS-C,^8^ or classical mucocutaneous Kawasaki disease findings.^9^ Additionally, our patient was fully vaccinated for COVID-19, which may have conferred protection against pulmonary disease but less so against her cutaneous immunopathogenesis. Although her COVID-19 strain was undetermined, infection with a less virulent yet persistent variant like Omicron BA.1, predominant in Southern California at the time, may have prompted greater immune complex formation. Concurrent asymptomatic streptococcal pharyngitis or gram-negative urinary tract infection, identified on the basis of laboratory testing, may have also contributed. Nevertheless, MIS-C remains a consideration given her cutaneous presentation and response to IVIG,^10^ particularly as MIS-C skin disease severity may inversely correlate with respiratory disease.^11^ Genetic and immune profiling in unusual COVID-19 presentations may 1 day yield additional insights.

Extensive scarring complicated our patient’s recovery, prompting laser therapy. Ablative lasers are used cautiously in autoimmune conditions due to concern for flares.^12^ Rare laser-induced vasculitic reactions are reported primarily after hair removal, not scar resurfacing or vascular lasers.^12^ Emerging evidence supports safe laser use in well-controlled autoimmune conditions, with pulsed-dye laser improving cutaneous lupus lesions and fractional ablative lasers aiding post-inflammatory sequelae of morphea.^13^^,^^14^

Data on laser usage after vasculitic conditions remain limited. In our patient, adjunct immunosuppression was maintained during laser initiation to mitigate potential inflammatory provocation, and marked scar improvement occurred without vasculitis recurrence. After a complicated course, the patient returned near her previous healthy baseline.

## Conflicts of interest

None disclosed.
